# Identification of cardiac malformations in mice lacking *Ptdsr *using a novel high-throughput magnetic resonance imaging technique

**DOI:** 10.1186/1471-213X-4-16

**Published:** 2004-12-22

**Authors:** Jürgen E Schneider, Jens Böse, Simon D Bamforth, Achim D Gruber, Carol Broadbent, Kieran Clarke, Stefan Neubauer, Andreas Lengeling, Shoumo Bhattacharya

**Affiliations:** 1 Department of Cardiovascular Medicine, University of Oxford, Wellcome Trust Centre for Human Genetics, Roosevelt Drive, Oxford, OX3 7BN UK; 2 German Research Centre for Biotechnology, Division of Microbiology, Junior Research Group Infection Genetics, Mascheroder Weg 1, 38124 Braunschweig, Germany; 3 Department of Pathology, School of Veterinary Medicine Hannover, Bünteweg 17, 30559 Hannover, Germany; 4 Department of Physiology, University of Oxford, Parks Road, Oxford OX1 3PT UK

## Abstract

**Background:**

Congenital heart defects are the leading non-infectious cause of death in children. Genetic studies in the mouse have been crucial to uncover new genes and signaling pathways associated with heart development and congenital heart disease. The identification of murine models of congenital cardiac malformations in high-throughput mutagenesis screens and in gene-targeted models is hindered by the opacity of the mouse embryo.

**Results:**

We developed and optimized a novel method for high-throughput multi-embryo magnetic resonance imaging (MRI). Using this approach we identified cardiac malformations in phosphatidylserine receptor (*Ptdsr*) deficient embryos. These included ventricular septal defects, double-outlet right ventricle, and hypoplasia of the pulmonary artery and thymus. These results indicate that *Ptdsr *plays a key role in cardiac development.

**Conclusions:**

Our novel multi-embryo MRI technique enables high-throughput identification of murine models for human congenital cardiopulmonary malformations at high spatial resolution. The technique can be easily adapted for mouse mutagenesis screens and, thus provides an important new tool for identifying new mouse models for human congenital heart diseases.

## Background

Congenital malformations are a major cause of death in childhood, and are typically characterized by lesions that do not compromise fetal survival. For instance, congenital heart disease (CHD) typically consists of lesions such as ventricular and atrial septal defects, which are compatible with fetal hemodynamics [1]. Although human genetic studies have identified some genes that cause congenital cardiac malformations, the molecular and developmental mechanisms underlying most of these defects remain largely unknown. Despite the high incidence of CHD (~1% of live births), only a handful of genes have been identified that when mutated, result in congenital heart disease [2-4].

The mouse is a particularly good model for studying mechanisms of cardiac diseases as its anatomy and development resembles that of the human more closely than any other genetically tractable organism. Importantly, the mouse is amenable to genotype-driven approaches such as transgenic knockouts of defined candidate genes [5], genome wide mutagenesis approaches using gene-trap [6] or transposon insertion screens [7], and high-throughput phenotype-driven screens that rely, for instance, on *N*-ethyl-*N*-nitrosourea (ENU) mutagenesis [8,9]. However, high-throughput cardiovascular genomic approaches in the mouse have been hampered by the paucity of phenotyping tools that allow efficient identification of complex cardiac malformations. As mouse embryos are opaque, late developmental defects are particularly difficult to identify. For instance, cardiac septal defects or outflow tract abnormalities can only be confidently identified after 14.5 days post coitum (dpc) when cardiac and outflow tract septation are completed in normal embryos. The identification of malformations in late gestation embryos typically relies on serial histological sectioning, which is extremely labor intensive. Furthermore, this often results in the irretrievable loss of 3D information, which is essential for the interpretation of complex cardiac malformations. In addition, standard pathological analysis is not amenable to high-throughput phenotype screening protocols that are required for any mutagenesis screen aiming at the functional dissection of the developmental biology of cardiac diseases. Therefore, new technological approaches must be harnessed that allow an efficient phenotyping of heart defects and also of subtle cardiac abnormalities that are at danger of being overseen in traditional histopathology screens. This is even more important in the light of upcoming new endeavors in functional mouse genome annotation [10]. Currently, new large-scale mouse mutagenesis screens are being set up in the US and in Europe that aim to produce heritable mutations in every gene in the mouse genome [11-13]. To make full use of these new mouse mutant resources more precise and efficient phenotyping methods are urgently needed [12-14]. The success of genome wide saturation mutagenesis screens depends therefore on improved phenotyping, and new high-resolution imaging approaches for mouse mutants are one of the most important which need to be established.

We previously reported the development of fast gradient-echo MRI of single mouse embryos [15-17]. This resulted in the acquisition of a 3D dataset in under 9 hours, with an experimental image resolution of 25 × 25 × 26 μm/voxel. We showed that MRI is capable of accurately identifying normal embryonal structures, and cardiac and adrenal malformations in knockout mouse embryos, and we have validated this technique by performing in depth histological examinations of imaged embryos [15-17]. These experiments showed that single-embryo MRI could correctly identify all cardiac lesions (atrial septal defects, ventricular septal defects, outflow tract defects such as double-outlet right ventricle, and aortic arch defects) except those under 20 μm – which is below the resolution of the MRI technique. As this method images single embryos in overnight runs, it still lacks the throughput required for phenotype-driven mutagenesis screens. For instance, in a typical recessive ENU mutagenesis screen, to screen 50 ENU mutant lines using a 3-generation breeding scheme would require the analysis of ~1200 embryos [18]. We now report the development of a method of imaging up to 32 embryos simultaneously in a single unattended overnight run, at high spatial resolution. Allowing ~30 minutes per embryo, the analysis of 1200 embryos would take 75 working days for a single trained individual. We show that this high-throughput multi-embryo MRI technique can be used to rapidly identify unsuspected embryonal cardiac and visceral malformations. Using this technique we could identify a novel, and hitherto unsuspected role for the phosphatidylserine receptor (*Ptdsr*) in controlling ventricular septal, outflow tract and pulmonary artery development. In addition, we found thymus hypoplasia in *Ptdsr*-deficient embryos. These findings suggest that a novel *Ptdsr*-mediated pathway is required for cardiac and thymus development.

## Results

### Multi-embryo imaging

**Figure 1:**
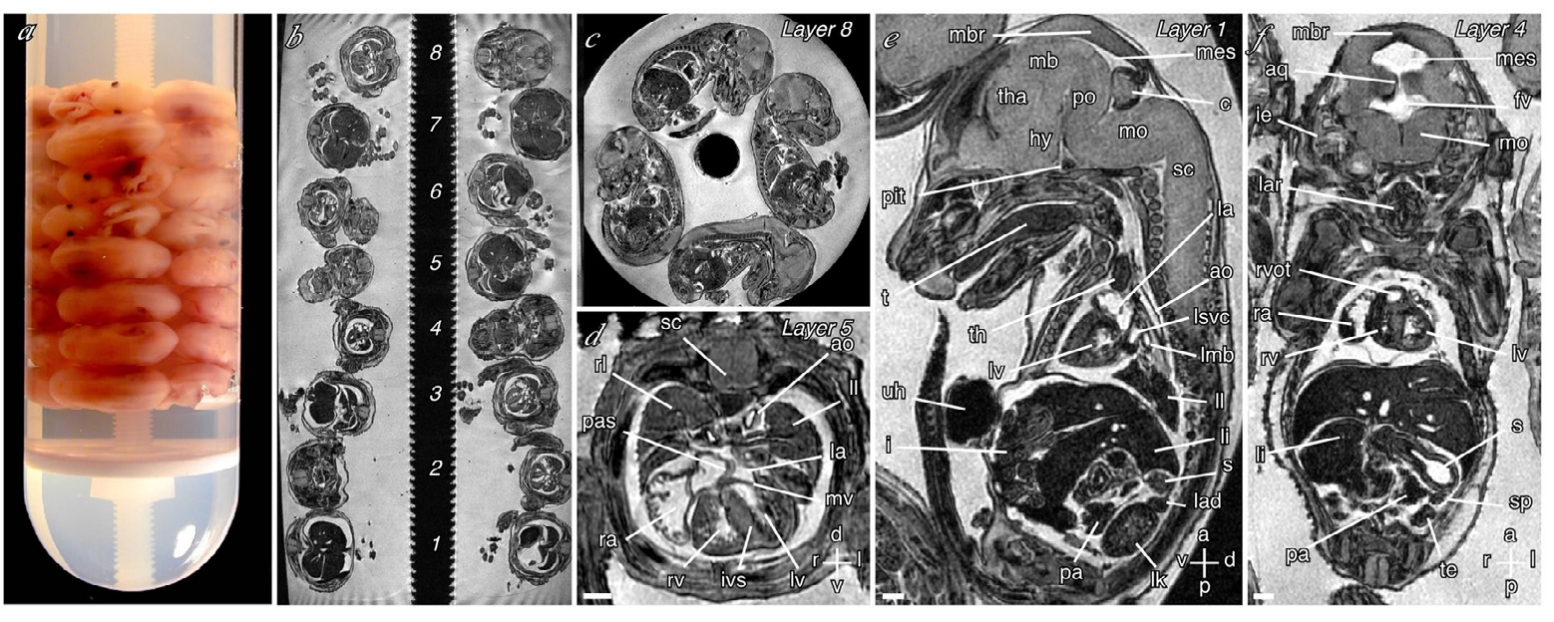
**High-throughput high-resolution magnetic resonance microscopy. **(**a**) Stack of 32 embryos embedded in a NMR tube. (**b**) Section through the long axis of the NMR tube showing embryos in eight layers. (**c**) Sagittal section through layer 8 showing the four embryos in this layer. (**d–f**) Transverse, sagittal, and coronal sections through individual embryos in layers 5, 1 and 4 respectively. The voxel size is 25.4 × 25.4 × 24.4 μm. Structures indicated are the spinal cord (sc), the right and left lungs, atria and ventricles (rl, ll, ra, la, rv, lv), primary atrial and interventricular septa (pas, ivs), mitral valve (mv), midbrain roof (mbr), midbrain (mb), mesencephalic vesicle (mes), thalamus (tha), hypothalamus (hy), pons (po), cerebellum (c), medulla oblongata (mo), pituitary (pit), tongue (t), thymus (th), left superior vena cava and main bronchus (lsvc, lmb), aorta (ao), liver (li), stomach (s), left adrenal and kidney (lad, lk), pancreas (pa), intestines (i), umbilical hernia (uh), aqueduct of Sylvius (aq), fourth ventricle (fv), inner ear (ie), larynx (lar), right ventricular outflow tract (rvot), spleen (sp), and testes (te). Scale bars = 500 μm; axes: d – dorsal; v – ventral; r – right; l – left; a – anterior, p – posterior.

We modified our previously described fast gradient echo magnetic resonance imaging technique [15-17] to image embryos embedded in four to eight layers (16 – 32 embryos total) in 28 mm nuclear magnetic resonance tubes using a single quadrature driven birdcage coil (Figure 1a). Preparation and embedding of embryos typically took less than an hour. In initial experiments we imaged up to 16 embryos simultaneously in overnight runs of <9 hours, with an experimental resolution of 51 × 51 × 39 μm. Subsequently, we used a custom made optimized probe with an increased sensitivity range to enhance imaging throughput. This allowed us to image 32 embryos simultaneously (Figure 1a,b), but with a larger matrix size and an increased field-of-view in the long axis of the tube. For these experiments we imaged embryos for ~12 hours, and achieved an improved experimental resolution of 43 × 43 × 36 μm. The optimized coil used for 32 embryos MRI (Figure 1) has a sensitivity range in z-direction of close to 50 mm. The artefacts seen at both ends in the longitudinal image (Figure 1b) are caused by B1-inhomogeneities at the end-rings of the coil. However, accurate image analysis for the entire data set remains possible if the height of the embryo stack does not exceed approximately 47 mm as demonstrated in the corresponding axial views of the top and the bottom layer (Figure 1c,d). The resolution achieved with the multi-embryo MRI technique allowed us to visualize the heart, cardiac septa, central nervous system, and visceral organs in fine detail (Figure 1d–f), in embryos taken from each layer. The data are permanently archived on DVDs, for subsequent analysis. Construction of 3D reconstructions of the heart typically takes ~4 hours, but was not necessary for the identification of cardiovascular defects.

### Sensitivity and specificity of multi-embryo imaging

**Figure 2:**
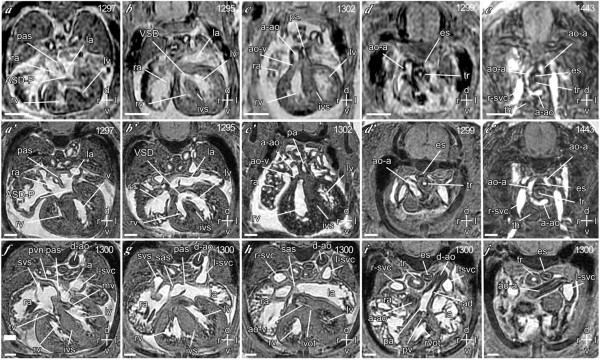
**Identification of septal, outflow tract, and aortic arch malformations using multi-embryo MRI **(**a – e'**) Images of transverse sections from 5 *Cited2*^-/- ^embryos obtained using the multi-embryo technique (*a–e*) compared with images from the same embryos obtained subsequently using the single embryo technique (**a'–e'**). (**a, a'**) Section showing left and right atria and ventricles (la, ram, live, rave). The atria are separated by the primary atria septum (pas), which is deficient at its ventral margin creating an osmium premium type of atria septal defect (ASD-P). (**b, b'**) Section showing a ventricular septal defect (VSD) in the interventricular septum (ivs). (**c, c'**) Section showing double outlet right ventricle, wherein the ascending aorta (a-ao) and the pulmonary artery (pa) both arise from the right ventricle (rv). The aortic valve (ao-v) is indicated. (**d, d'**) Section showing a right-sided aortic arch (ao-a) passing to the right of the trachea (tr) and the esophagus (es). (**e, e'**) Section showing bilateral aortic arches (ao-a) forming a vascular ring around the trachea (tr) and the esophagus (es). Also indicated are the thymus (th) and the right superior vena cava (r-svc). (**f – j**) Serial transverse sections through a wild-type heart obtained using single embryo MRI, demonstrating corresponding normal structures, including the systemic venous sinus (svs), left superior vena cava (l-svc), pulmonary vein (pvn), descending aorta (d-ao), mitral and tricuspid valves (mv, tv), the secondary atrial septum (sas), left and right ventricular outflow tracts (lvot, rvot), pulmonary valve (pv), and arterial duct (ad) of the pulmonary artery. Scale bars = 635 μm for multi-embryo, and 317 μm for single embryo images; axes: d – dorsal; v – ventral; r – right; l – left.

**Figure 3:**
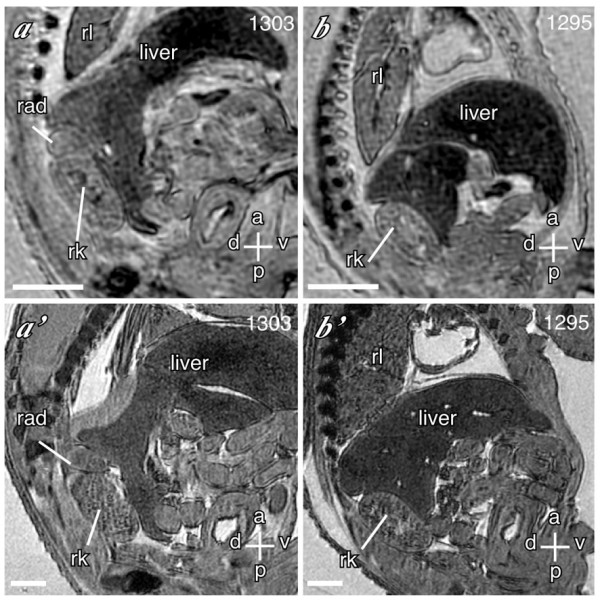
**Identification of adrenal agenesis using multi-embryo MRI **Images of coronal sections from 2 embryos obtained using the multi-embryo technique (**a, b**) compared with images from the same embryos obtained subsequently using the single embryo technique (**a', b'**). (**a, a'**) Normal right adrenal gland (rad) anterior to the right kidney (rk) in a wild-type embryo. The right lung (rl) is indicated. (**b, b'**) Agenesis of right adrenal gland in a *Cited2*^-/- ^embryo. Scale bars = 635 μm for multi-embryo, and 317 μm for single embryo images; axes: d – dorsal; v – ventral; a – anterior, p – posterior.

To assess the sensitivity and specificity of multi-embryo imaging in comparison to single-embryo imaging, we used a model of *Cited2 *deficiency [19]. Embryos lacking *Cited2 *(*Cited2*^-/-^) have diverse cardiac malformations, including atrial and ventricular septal defects, outflow tract and aortic arch malformations, and adrenal agenesis [15,17,19]. As the *Trp53*-repressor gene *Cdkn2a*^*P19ARF *^is a target of *Cited2 *[20], we also examined embryos lacking both *Cited2 *and *Trp53 *to determine if this would rescue the heart and adrenal defects in *Cited2*^-/- ^mice. We imaged 50 embryos using the multi-embryo technique in the 16-embryo mode. Embryonal genotypes included 12 wild-type, 13 *Cited2*^+/-^, 14 *Cited2*^-/-^, three *Cited2*^-/-^: *Trp53*^-/-^, four *Cited2*^-/-^: *Trp53*^+/-^, two *Trp53*^+/-^, and two *Trp53*^-/-^. We analyzed the data from each embryo for cardiac malformations without knowledge of the genotype. This typically took a maximum of 30 minutes per embryo. We scored each embryo for atrial and ventricular septal defects (ASD, VSD), outflow tract (e.g. double-outlet right ventricle, common arterial trunk), and aortic arch malformations (e.g. right-sided or bilateral aortic arch, retroesophageal subclavian artery), and for adrenal agenesis. Each embryo was then re-imaged singly at high resolution, and the data re-analyzed as before. In this group we identified 20 embryos with ASD, 19 with VSD, 18 with outflow tract defects, 11 with aortic arch defects, and 21 with bilateral adrenal agenesis, using high-resolution single embryo imaging. In comparison to single embryo imaging, the overall sensitivity and specificity of multi-embryo imaging for cardiac malformations was 88% and 92% respectively. For ASD (18 identified by single embryo imaging) the sensitivity and specificity was 85% and 95%; for VSD 94% and 94%; for outflow tract malformations 94% and 100%; and for aortic arch malformations 91% and 100% respectively (Figure 2). For bilateral adrenal agenesis, the sensitivity was 100% and specificity was 95% (Figure 3). Embryos lacking both *Cited2 *and *Trp53 *had cardiovascular defects and adrenal agenesis, indicating that *Trp53 *does not play a major role in the genesis of these defects in mice lacking *Cited2*. These results indicate that multi-embryo MRI is a potentially powerful high-throughput tool for efficiently characterizing cardiovascular malformations and identifying other defects in organogenesis.

### Cardiac malformations in mice lacking *Ptdsr*

**Figure 4:**
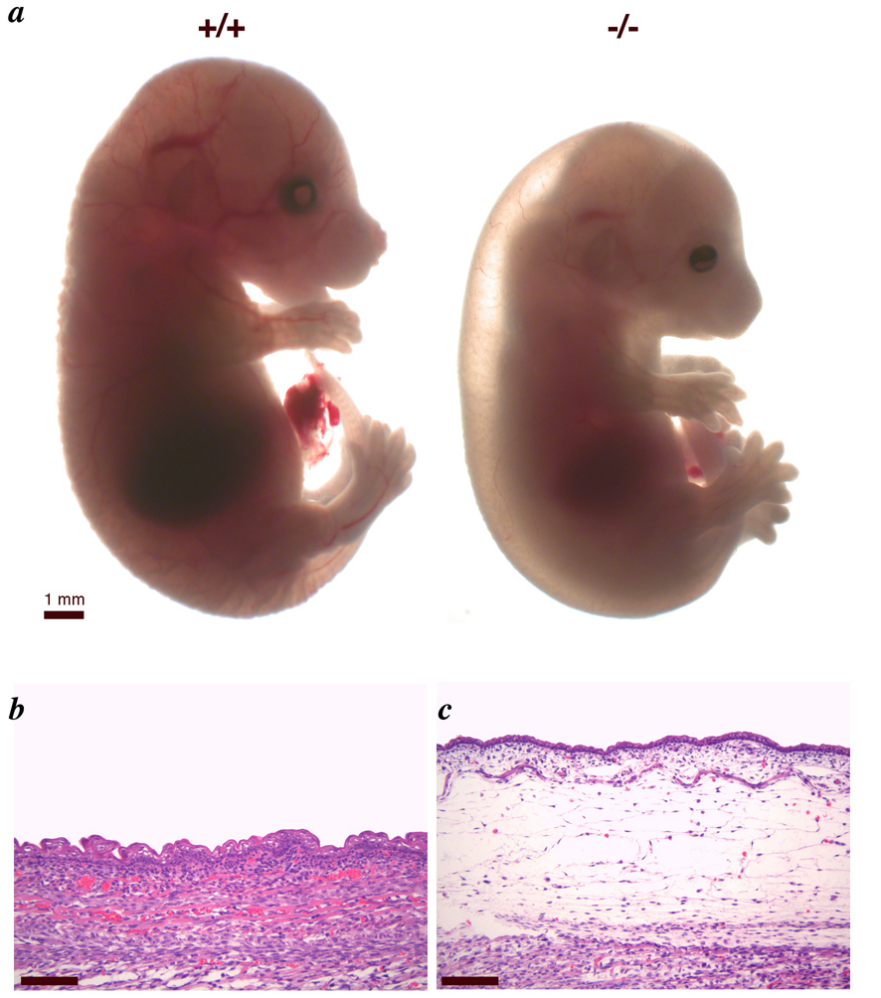
**Edema in *Ptdsr*^-/- ^mice **(**a**) The *Ptdsr*^-/- ^mutant (15.5 dpc) is growth retarded and the severe edema along the back of the embryo is visible. (**b, c**) Sagital sections of embryos at 16.5 dpc. The mutant embryo (c) exhibits massive subcutaneous edema compared to a wild-type (b) littermate. Scale bar = 100 μm in (b) and (c).

**Figure 5:**
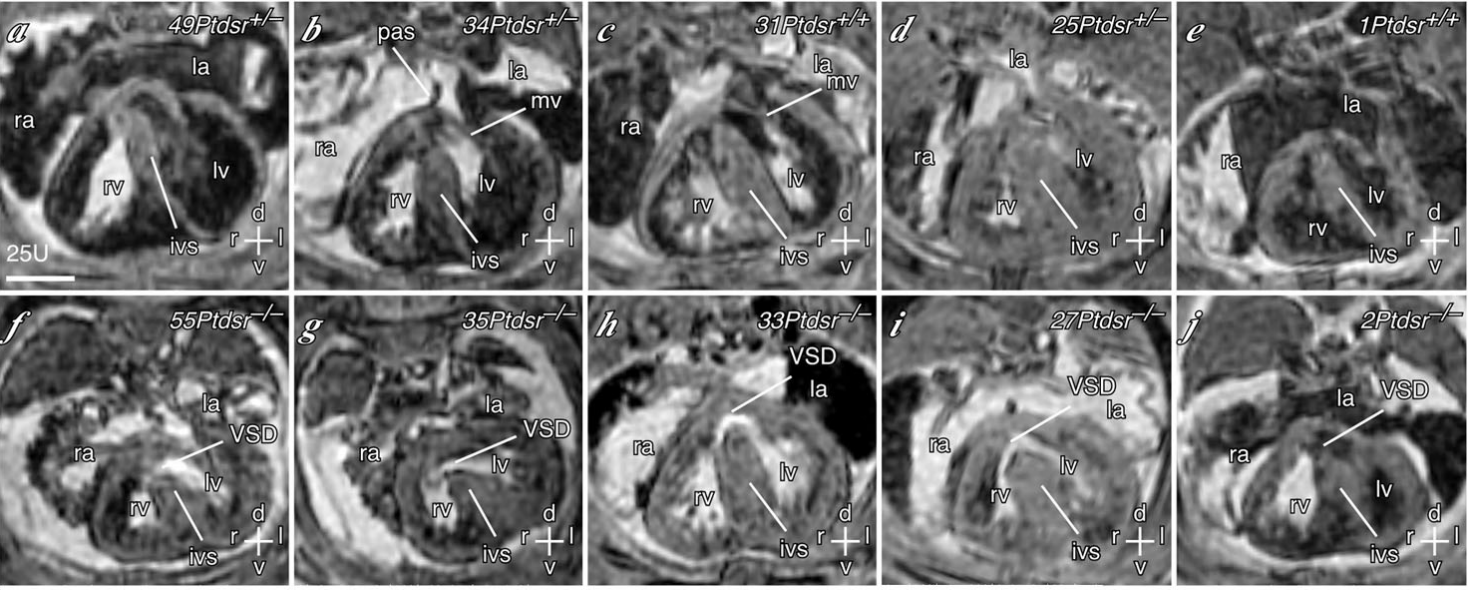
**Identification of cardiac malformations in *Ptdsr*^-/- ^embryos using multi-embryo MRI **(**a–e**) Transverse thoracic sections showing the heart of heterozygous or wild-type control embryos from each litter. The left and right ventricles (lv, rv) are separated by the interventricular septum (ivs). The left and right atria (la, ra) are also indicated, separated by the primary atrial septum (pas). (**f–i**) Corresponding sections through littermate *Ptdsr*^-/- ^embryos, showing ventricular septal defects (VSD). Scale bar = 635 μm; axes: d – dorsal; v – ventral; r – right; l – left; a – anterior, p – posterior. Individual embryos are indicated by number.

**Figure 6:**
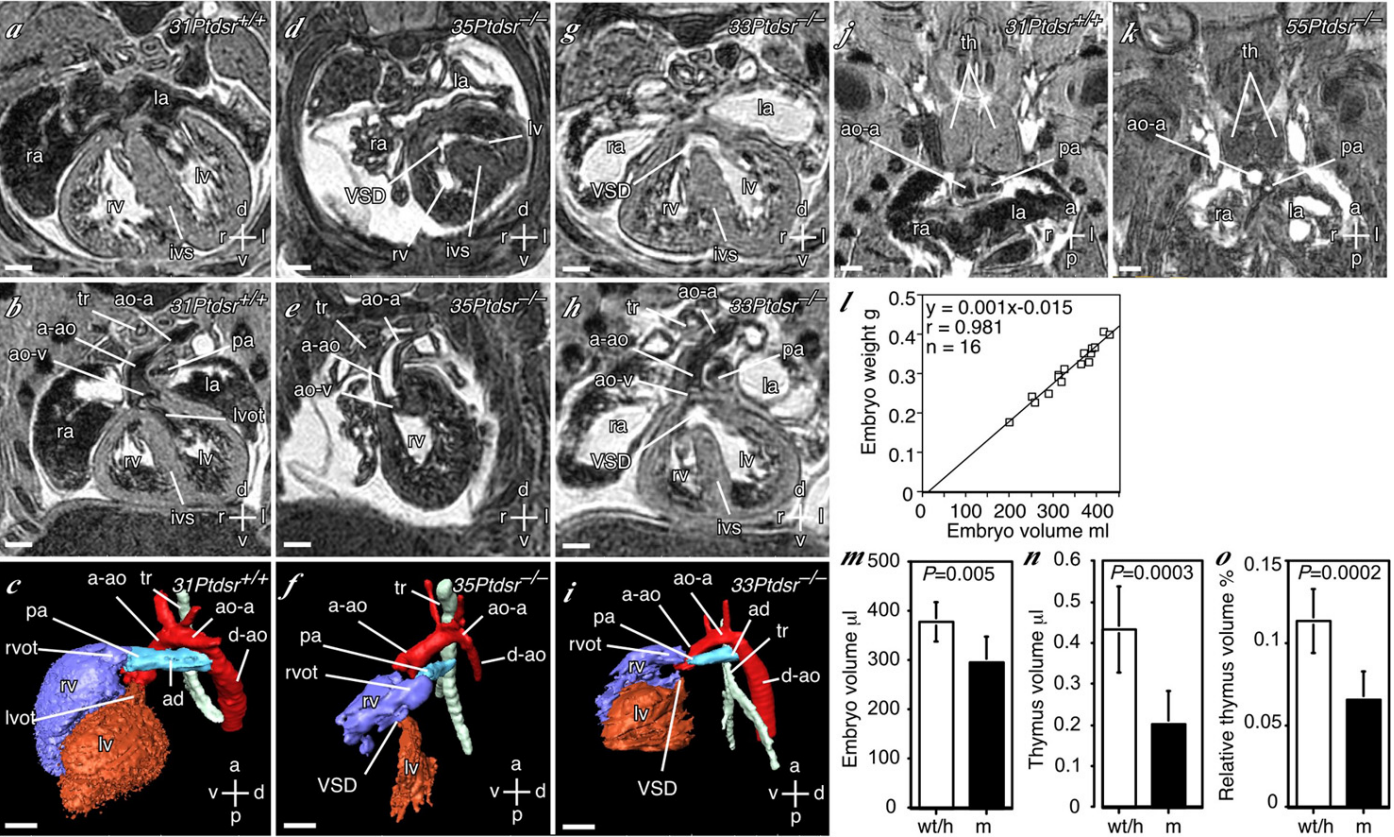
**Cardiac malformations and thymus hypoplasia in *Ptdsr*^-/- ^embryos**. (**a–c**) Transverse and oblique (through the plane of the ascending aorta) sections, and 3D reconstruction (left-ventral oblique view) of a heart of a wild-type embryo at 15.5 dpc. The left and right ventricles (lv, rv) are separated by the interventricular septum (ivs). The left and right atria (la, ra), and the trachea (tr) are also indicated. The ascending aorta (a-ao) arises from the left ventricular outflow tract (lvot), via the aortic valve (ao-v), and continues on as the aortic arch (ao-a), which joins the descending aorta (d-ao). The pulmonary artery (pa) arises from the right ventricular outflow tract (rvot), and continues as the arterial duct (ad), which joins the descending aorta. (**d–f**) Corresponding images of a *Ptdsr*^-/- ^embryo, showing a smaller heart with a ventricular septal defect (VSD). The aorta arises from the right ventricle. The pulmonary artery is small and its connection to the descending aorta (arterial duct) could not be identified. (**g–i**) Corresponding images of another *Ptdsr*^-/- ^embryo, showing a ventricular septal defect (VSD). The aorta overrides the VSD resulting in a double-outlet right ventricle. (**j, k**) Coronal sections of *Ptdsr*^+/+ ^and *Ptdsr*^-/- ^embryos, showing the two lobes of the thymus (th). The arterial duct of the pulmonary artery in the *Ptdsr*^-/- ^embryo is narrowed. (**l**) Correlation between embryo weight and volume. Scattergram of embryo weight versus embryo volume measured from multi-embryo MRI datasets for 16 embryos using Amira. The co-efficient of regression (r) is indicated. (**m, n**,) Absolute embryo and thymus volumes (μl) were measured from the MRI datasets from 5 wild-type (wt), 3 heterozygote (h), and 8 *Ptdsr*^-/- ^(m) embryos at 15.5 dpc. There was no significant difference in the wild-type and heterozygote data, which were therefore pooled together (wt/h). (***o***) Relative thymus volumes (% of embryo volume) were calculated as *Ptdsr*^-/- ^embryos were slightly smaller than littermate wild-type embryos. The data are represented as mean ± S.D. The probability of a type I error (*P*) is indicated. Scale bars = 317 μm; axes: r – right; l – left; d – dorsal; v – ventral; a – anterior, p – posterior. Individual embryos are indicated by number.

**Figure 7:**
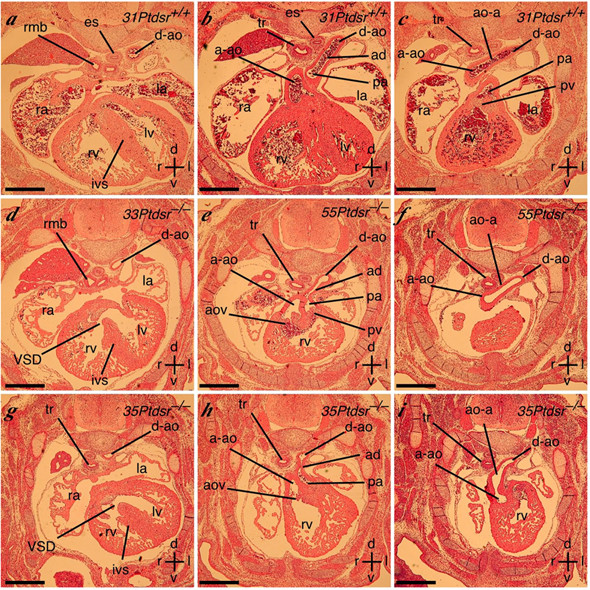
**Cardiac malformations in *Ptdsr*^-/- ^embryos: analysis using histology **Embryos analyzed by MRI (Figure 3) were sectioned transversely and stained with hematoxylin and eosin. **(a–c) **Serial caudal to cranial sections of the wild-type embryo showing normal cardiac and vascular anatomy. The left and right ventricles (lv, rv) are separated by the interventricular septum (ivs). The ascending aorta (a-ao) arises from the left ventricle, continues on as the aortic arch (ao-a), which joins the descending aorta (d-ao). The pulmonary artery (pa) arises from the right ventricle via the pulmonary valve (pv) and continues as the arterial duct (ad), which joins the descending aorta. The left and right atria (la, ra), trachea (tr), right main bronchus (rmb) and esophagus (es) are indicated. **(d) **Section through embryo 33 indicating the ventricular septal defect (VSD). **(e, f) **Sections through embryo 55 showing that both aorta and pulmonary artery arise from the right ventricle (double outlet right ventricle), and that the arterial duct of the pulmonary artery is narrow in comparison to the aorta – indicating pulmonary artery hypoplasia. The aortic valve (aov) is indicated. **(g–i) **Serial caudal to cranial sections through embryo 35 showing a VSD, aorta arising from the right ventricle (double outlet right ventricle), and a severely narrowed arterial duct. Scale bars = 500 μm; axes: r – right; l – left; d – dorsal; v – ventral. Individual embryos are indicated by number.

We next evaluated the role of multi-embryo MRI in analyzing unexplained lethality in embryos generated in collaborating laboratories. Recently, we have generated mice lacking the *phosphatidylserine receptor *(*Ptdsr*^-/-^) on a C57BL/6J background, by gene targeting in embryonic stem cells [21]. Ptdsr is a nuclear protein of unknown function, which is essential for the development and differentiation of multiple organs during embryogenesis [21-24]. Ablation of *Ptdsr *function in knockout mice causes perinatal lethality, growth retardation [21,22,24] and a delay in terminal differentiation of the kidney, intestine, liver and lungs during embryogenesis [21]. In addition, *Ptdsr*^-/- ^embryos develop complex ocular lesions [21] as well as haematopoietic defects [24]. However, as many malformations have been described in *Ptdsr *mutants, none of those detected could explain the observed perinatal lethality of *Ptdsr*^-/- ^mice. In the process of phenotypical characterization of our *Ptdsr*-deficient mouse line, we frequently observed subcutaneous edema of varying sizes in *Ptdsr*^-/- ^embryos by gross inspection ([21] and Figure 4). As the development of edema in various mouse mutants is frequently associated with cardiovascular defects [25] we started to investigate if this also holds true for *Ptdsr*-deficient mice. We examined 8 embryos lacking *Ptdsr*, and 8 littermate wild-type or heterozygous controls using multi-embryo MRI. We found that 5 of 8 *Ptdsr*^-/- ^embryos had cardiac malformations, which included ventricular septal defects, double outlet right ventricle, and pulmonary artery hypoplasia (Figure 5). None of the wild-type or *Ptdsr*^+/- ^embryos had cardiac malformations. These findings were confirmed on single embryo imaging (Figure 6). Furthermore, to verify the identified cardiac defects in the *Ptdsr*^-/- ^mice we performed serial transverse sectioning of all analyzed embryos. In all cases, we could recognize again the same heart defects that were identified before using the multi-embryo MRI technique (Figure 7). In addition, we analyzed by multi-embryo MRI a second *Ptdsr*-knockout mouse line (*Ptdsr*^*tm1.1 Gbf*^), which is identical to the initially analyzed *Ptdsr *mutant except that the *loxP*-flanked neomycin selection cassette was removed by breeding the original *Ptdsr*^*tm1 Gbf *^knockout line [21] to a *CMV-Cre *deleter mouse line [26]. From this *Ptdsr*^*tm1.1 Gbf *^knockout mouse line we analyzed 8 *Ptdsr*^-/- ^embryos, and as littermate controls, 3 *Ptdsr*^+/+ ^and 1 *Ptdsr*^+/- ^embryos. We found ventricular septal defects in five out of the eight *Ptdsr*^-/- ^embryos (data not shown). Again we found no evidence for cardiac malformations in wild-type or heterozygous littermate control embryos.

**Figure 8:**
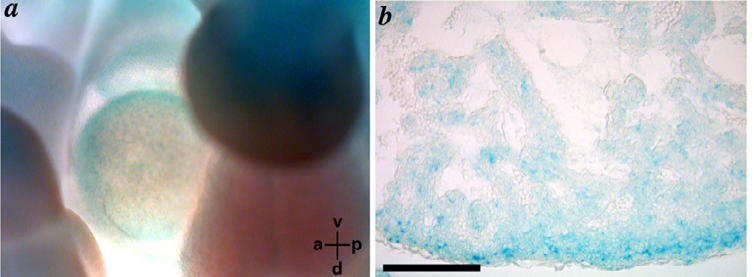
**Analysis of *Ptdsr *expression in the embryonic heart **(**a, b**) Staining of heterozygous *Ptdsr-βgeo*-embryos [21] using X-Gal at 10.5 dpc (a) and 11.5 dpc (b). **(a) **At 10.5 dpc *Ptdsr *expression can be seen throughout the heart. **(b) **Transverse sections of X-Gal stained embryos at 11.5 dpc showed an increased expression of *Ptdsr *in the myocardial wall and a beginning decrease of the expression in the trabeculation. Scale bar = 100 μm in (b).

**Figure 9:**
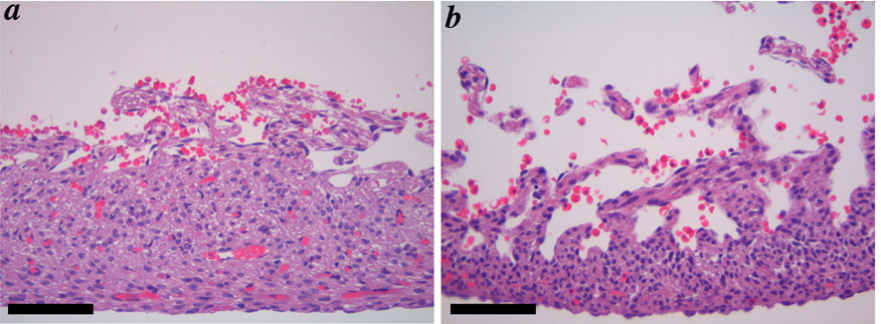
**Myocardial wall malformations in *Ptdsr*^-/- ^embryos **(**a, b**) Sagital sections of wild-type (a) and homozygous mutant (b) embryos at 16.5 dpc revealed a thinning of the myocardial wall (compact zone) and an increased myocardial trabeculation (b) in the mutant heart. Scale bar = 100 μm.

We also observed a modest degree of thymic hypoplasia in *Ptdsr*^-/- ^embryos (Figure 6k). To confirm this, thymus and embryo volumes were measured in 8 *Ptdsr*^-/- ^embryos and 8 wild-type or heterozygous control embryos at 15.5 dpc. Embryo volume measured from the MRI datasets correlated very strongly with embryo weight (Figure 6l), and was modestly reduced in *Ptdsr*^-/- ^embryos (Figure 6m). The volume of the thymus was significantly reduced in *Ptdsr*^-/- ^embryos, even after correction for embryo volume, to 58% of the control value (Figure 6n,o). To correlate the identified cardiopulmonary malformations in *Ptdsr*^-/- ^embryos with expression of the *Ptdsr *gene during heart development, we made use of our *Ptdsr *gene-trap reporter mouse line [21]. Using X-Gal staining in heterozygous embryos staged between 9.5 dpc and 12.5 dpc we found specific *Ptdsr *expression in the heart starting at 10.5 dpc and getting more defined to the compact zone and the trabeculi from 11.5 dpc onwards (Figure 8). Furthermore, when we analyzed *Ptdsr*^-/- ^hearts by histopathology at 16.5 dpc we observed a severe differentiation defect in the compact zone as well as in the trabeculi (Figure 9), thus demonstrating that *Ptdsr *is in addition required for heart muscle differentiation at later stages of development. Taken together these results indicate that *Ptdsr *plays a hitherto unsuspected role in cardiovascular development as well as in cardiac muscle differentiation.

## Discussion

### Utility of MRI in identifying mouse models of human malformations

Our results show that it is possible to efficiently identify and quantitate relatively subtle cardiac and visceral malformations in late gestation mouse embryos using multi-embryo MRI. We have deliberately optimized our technique at late gestation in order to identify those congenital defects that allow survival through most of gestation. Importantly, these defects resemble human congenital malformations, and would provide mouse models for the study of these diseases. Our method represents a simpler alternative to the multi-coil approach published recently [27,28] in which up to eight fixed mouse embryos were imaged simultaneously, with a resolution of 200 μm. In comparison, the multi-embryo method described here has a higher experimental resolution of 43 μm. Notably, it requires substantial developmental and financial effort to equip an experimental MR-system with multiple coil and receive capability.

### Role of MRI in investigating murine embryonal or perinatal lethality

Many mouse gene knockouts display late gestational lethality, but incomplete analysis and loss of 3D information consequent to histological sectioning, results in major developmental malformations being frequently missed. As shown here, *Ptdsr*^-/- ^embryos on a C57BL/6J background develop heart defects. This was not noted in recent reports [22,24], and emphasizes the need not only for completely examining several mutant embryos, but also repeating these examinations in different genetic backgrounds. A major advantage of MRI is the ability to easily ship fixed embryos from referring laboratories to the laboratory that performs the MRI analysis. This minimizes the expense of animal relocation, re-derivation, and breeding required to generate the embryos, and significantly reduces animal experimentation.

### Role of MRI in high-throughput phenotype driven screens

MRI screens performed at late gestation would be expected to identify genes that affect later aspects of development, and identify hypomorphic and haploinsufficient alleles of genes that affect earlier steps of development. Published data from genome-wide ENU mutagenesis screens in the mouse indicate that ~30% progeny carry a heritable recessive phenotype, making 3-generation recessive screens the method of choice for identifying developmental malformations [18]. At least 24 3^rd ^generation progeny per 1^st ^generation mutant are typically screened, resulting in a >78% probability of identifying at least one fully penetrant recessive homozygous mutant. A typical recessive screen (e.g. 50 – 100 first generation mutants per year) would require the analysis of ~1200 – 2400 embryos per year. Our results show that multi-embryo MRI is eminently suitable for such throughput. Although, screening of 32 embryos overnight requires typically about 16 hours of data analysis, multi-embryo MRI mutagenesis screens can be easily performed at a reasonable scale if multiple operators analyze the data in parallel. As the data are permanently stored on DVDs, and can be analyzed easily by commercially available software, they can be without difficulty disseminated to specialists for further and more detailed analysis. Another powerful application of multi-embryo MRI will likely be the investigation and screening of potentially teratogenic drugs.

### Functional role of *Ptdsr *in heart development

Our results presented here indicate a new, and hitherto unsuspected role for the phosphatidylserine receptor in controlling ventricular septal, outflow tract, pulmonary artery, and thymus development. This finding suggests that a novel *Ptdsr*-mediated pathway is required for cardiac and thymus development. Recently, we have demonstrated that in contrast to previously reported hypothetical Ptdsr functions, the Ptdsr protein is not required for the clearance of apoptotic cells [21]. Moreover, detailed analysis of apoptosis induction and apoptotic cell clearance in *Ptdsr*^+/+ ^and *Ptdsr*^-/- ^embryos during heart development did not reveal any difference in the number and location of apoptotic cells between the genotypes (J.B., A.D.G. and A.L. unpublished observations). This further excludes that Ptdsr has any function in apoptotic cell clearance and points to other developmental mechanisms that are affected by *Ptdsr *ablation. The neural crest plays an important role in the development of the cardiac outflow tract, aortic arches, and the thymus [29]. As *Ptdsr*-deficient embryos lack intestinal ganglia [21] which are also derived from the neural crest, these results suggest that *Ptdsr*^-/- ^mice may have an underlying neural crest defect. Importantly, dysfunction of these *Ptdsr*-mediated pathways during development could also potentially result in heart defects in humans.

## Conclusions

Our results validate the utility of multi-embryo MRI for high-throughput identification of murine models for human congenital cardiac malformations, and using this technique we have shown that *Ptdsr *is essential for normal cardiac development. Further experiments are needed to define exactly in which pathways *Ptdsr *is involved during heart development. We expect that multi-embryo MRI will be an important technology for future phenotype-driven mouse mutagenesis screens. The technology can be easily implemented at standard MRI imaging centers, thus allowing by collaboration with individual researchers or mouse mutagenesis centers, a high-throughput functional genetic dissection of mechanisms underlying cardiac development and congenital heart diseases.

## Methods

### Mice

*Cited2*^-/- ^[19], *Trp53*^-/- ^[30], and *Ptdsr*^-/- ^mice [21] have been described previously. All embryos were harvested at 15 days after detection of the vaginal plug.

### Embryo preparation

Embryos were fixed in 4% paraformaldehyde at 4°C for ~1 week, and then embedded in 1% agarose (Seakem) containing 2 mM gadolinium-diethylenetriamine pentaacetic anhydride (Gd-DTPA, Magnevist, Schering UK) in 28 mm nuclear magnetic resonance tubes (Figure 1). The left forelimb was removed from each embryo to facilitate the identification of the left side. In addition, embryos had other limbs and/or tails removed before embedding so that each embryo in a given layer (of four embryos) could be unequivocally identified.

### Magnetic resonance imaging

Single embryo imaging was performed as described previously [15-17]. For multi-embryo imaging, we used the same 11.7 Tesla (500 MHz) vertical magnet (Magnex Scientific, Oxon, UK). This was interfaced to a Bruker Avance console (Bruker Medical, Ettlingen, Germany) equipped with a shielded gradient system with a maximal gradient strength of 548 mTesla/m (Magnex Scientific, Oxon, UK), and quadrature-driven birdcage type coils with an inner diameter of 28 mm (Rapid Biomedical, Würzburg, Germany). Compressed air at room temperature was used to reduce the heating induced by the gradients. A 3D spoiled gradient echo sequence (echo time 10 ms), a π/2 excitation pulse with rectangular pulse shape, (π/2 = 100 μs), was used with a short repetition time (30 ms) to obtain strong T_1 _contrast. A matrix size of 512 × 512 × 768 (bandwidth: 130 Hz/pixel) at a field of view of 26 × 26 × 30 mm achieved an experimental resolution of 51 × 51 × 39 μm when imaging up to 16 specimens. In case of 32 embryos, a matrix size of 608 × 608 × 1408 at field of view of 26 × 26 × 50 mm, yielded an experimental resolution of 43 × 43 × 36 μm. The total experimental time was ~8.75 hours for 16 embryos, and ~12.3 hours for 32 embryos (typically overnight runs) whereby each phase encoding step was averaged four times.

### Data reconstruction and analysis

The raw MR data were reconstructed into a stack of 1024 (for 16 embryos), or 2048 (for 32 embryos) 2D TIFF files (16 bit pixel resolution, 2 or 4 GB total size) using purpose-written software as described previously [16]. The TIFF files were analyzed using Amira 3.1(TGS Europe, Mérignac Cedex, France). 3D reconstructions were performed using the Image Segmentation Editor, and tissue volumes for morphometric analysis were measured using the Measure Tissue Statistics tool available in Amira 3.1. The probability (p) of a Type I error was calculated using a 2-sample equal variance 2-tailed *t*-test in Microsoft Excel.

### Histology

Embryos were dehydrated in ethanol, embedded in paraffin wax, and sections were stained with hematoxylin and eosin.

### X-Gal staining of embryos

Embryos were dissected free of extraembryonic membranes and then fixed in 4% paraformaldehyde at 4°C. Expression of *Ptdsr *was detected by staining the embryos overnight in X-Gal according to standard protocols. The embryos were postfixed in 4% paraformaldehyde and processed for documentation or histology.

## Authors' contribution

J.E.S. developed the multi-embryo MRI technique, J.B. generated both *Ptdsr *knockout lines and harvested embryos for MRI and histopathological analysis, S.D.B developed the sample preparation for embryonic MRI, A.D.G. carried out the histopathological analysis of *Ptdsr*^-/- ^mutants, C.B. prepared the embryos for MRI, K.C. and S.N. assisted the experimental development, S.B. analyzed the MRI and histopathological data, A.L. and S.B. were responsible for the co-ordination of the study and the drafting of the paper.
